# Examining Individuals’ Use of the Internet for Health Care Activities Over Time: Results from the US National Health Interview Survey

**DOI:** 10.2196/58362

**Published:** 2025-02-26

**Authors:** Zachary Junkins, Nusrath Zahan, David Neyens

**Affiliations:** 1 Department of Industrial Engineering Clemson University Clemson, SC United States

**Keywords:** internet, web search, internet search, internet use, searching behavior, access to health information, telemedicine, telehealth, virtual care, virtual health, virtual medicine, logistic regression model, regression model, National Health Interview Survey, NHIS

## Abstract

**Background:**

Telehealth is an increasingly important component of health care services. Telehealth services may present an opportunity to increase the equity, accessibility, and effectiveness of health care. As such, it is critical that telehealth design focuses on reducing the barriers to access and usability that may impair some telehealth users.

**Objective:**

Our goal was to identify different demographic characteristics, behaviors, or opinions that may predict groups who are likely to face a barrier to using telehealth services.

**Methods:**

We used data from the National Health Interview Survey and multiple logit regression models focused on different aspects of telehealth to examine three different avenues of telehealth service: looking up health information using the internet, scheduling an appointment using the internet, and communicating with a care provider through email using the internet in order to consider the ways in which different telehealth services may face different barriers.

**Results:**

Our results suggest that middle-aged (36-55 years old) and older adult (56-85 years old) respondents were significantly less likely to look up health information using the internet or schedule an appointment using the internet versus younger individuals (18-35 years old). Specifically, our analysis found that middle-aged adults were found to have a higher odds ratio than older adults (0.83 vs 0.65) for looking up health information using the internet. We also found that there were differences in age groups for using technology to perform health care–related tasks. In terms of searching for health information using the internet and scheduling appointments using the internet, we found differences between men and women, with women being significantly more likely than men to look up health information using the internet, schedule an appointment using the internet, and communicate with a care provider through email using the internet. Across all the investigated variables, we found that the rates of using the internet for looking up health information, scheduling an appointment, and communicating with a care provider over email increased substantially across the study period. The impact of costs was inconsistent across the different models in our analysis. We also found that there is a strong correlation between respondents’ collaboration in their personal health and the likelihood that they would use telehealth services to meet these needs.

**Conclusions:**

This analysis provides an exploratory look at the data to highlight barriers that may impact a user’s ability to access telehealth services in the context of other potential predictor variables to account for the real-world variability that these may present. Future work should examine the complex relationships of those variables and understand how these interactions are correlated with the respondents’ use of telehealth.

## Introduction

### Background

With the advancement of digital technologies, the use of technology in health care has grown [1,2]. While there have been several attempts to better understand the use of digital technologies in health care, much of the work has been done in silos and is sometimes limited in scope [2,3]. Generally, telehealth refers to health care communication through technology, often used in conjunction with telemedicine and eHealth [3-5]. Mobile health is an additional term that describes telehealth services in the context of mobile devices [6]. Telehealth is increasingly being used in growing populations, offering distinct advantages and potential barriers for different patient populations and their care.

### Benefits Associated With Telehealth

The increased adoption of telehealth services has allowed for its impacts to be examined on actual patients receiving telehealth care [7]. It has been shown that when telehealth is implemented with current best practices, it may help to balance the health care supply and demand disparity [8], improve patient access to care [8,9], and reduce the cost of care [8,9]. Due to increasing demand with changes in population sizes and demographics, as well as decreasing supply as care providers retire or change careers [8], telehealth services and telehealth programs have helped bridge this gap [10,11]. Telehealth also has been shown to improve patients’ ability to access care, thus enabling patients to receive efficient and cost-effective care [8,10] by reducing the impacts of geographical barriers [12] and cost barriers to care [13]. Telehealth may also improve the cost-effectiveness of care, with [14] finding that the “all-cause” cost of providing care to older patients (older than 65 years) decreased from US $937.25 to US $491.52 with the inclusion of telehealth services. Telehealth can also offer support for individuals seeking mental health care where mental health specialties are distant or when there may be patient privacy issues in obtaining mental health care [15].

### Barriers to the Use of Telehealth Services

There are several potential barriers that have been identified related to the use and effective implementation of telehealth services [7]. A notable barrier to telehealth services is patients’ health literacy [16-18]. Individuals with lower levels of health literacy have shown lower comfort levels with using telehealth technologies [17,19]. Older people are using as well as providing telehealth services [20]. Yet, it has been suggested that older patients (older than 60 years) might have greater difficulty using telehealth services [21]. Additionally, technological infrastructure issues (low-quality, limited internet access) may reduce the ability of individuals to access telehealth services [22]. Individuals’ access to electronic devices (ie, smartphones, computers, and tablets) may also be a barrier to engaging in telehealth services [22]. In addition to the access issues, the time and costs required to engage in the services (both for the patients as well as clinicians) may be a barrier to rolling out large programs or integrating them into the workflow processes for clinical staff [23].

### Changes in Health Care Populations Over Time

There are several factors that have been shown to impact the use of web-based resources for health care and other telehealth services [24]. A 2018 study suggests that demographic factors, such as living in rural areas, age, and insurance types, impact telehealth implementation and use in health care facilities [24]. There have also been racial and ethnic discrepancies reported in the literature, with Black and Hispanic patients preferring emergency departments to telemedicine compared to White patients [25]. Age is also an important factor as the use of telehealth differs among different age groups [14,25]. In addition, health insurance access, and having internet access also influence the use of telehealth [14].

It is important to consider how user needs with telehealth change over time [26]. Changing population demographics, such as age, have major impacts on the use and function of health care [27]. Other additional factors, such as socioeconomic factors and changes in the prevalence of different health conditions, also impact the function of the health care system [27]. Over time, population demographics, access, and technology can change significantly, leading to incorrect conclusions or missed critical trends due to observing only one moment. It is important to consider all these elements when examining which predictors may indicate barriers to telehealth access. The objective of this research is to examine how individuals used the internet to support their health care over 7 years (2012-2018) using the National Health Interview Survey (NHIS) data. Specifically, we examined how different demographic variables and respondents’ perspectives influenced how individuals looked up health information using the internet, scheduled appointments using the internet, and emailed to communicate with a care provider using the internet.

## Methods

### Ethical Considerations

An ethics board review was not required for this analysis as this study used publicly available data.

### Overview

Each year the Centers for Disease Control and Prevention (CDC), a government agency assigned to monitor the general public’s behavior and health status conducts the NHIS, which is an annual cross-sectional survey designed to gather data on a variety of health-related topics throughout the United States with oversampling of certain demographic groups in a way that is nationally representative [28]. The survey weighting variable is poststratified based on the US Census data to represent national population characteristics [28]. These survey weights are identified for all combinations of persons within the variable WTFA_SA in the NHIS data. Due to the complex survey sampling strategy, the survey person weights must be used in analyzing the data to avoid substantial bias in the results [28].

For this analysis, the NHIS sample adult files for each year from 2012 to 2018 were used. For the files from 2012 to 2014, the American Standard Code for Information Interchange data file was combined using the stringr, stringi, foreign, and RCurl packages in R (R Foundation for Statistical Computing) to run the associated Statistical Analysis System statements to produce a .csv version of the sample adult file. For the years 2015-2018, a csv file was provided for the sample adult survey data. We created a single file by combining responses for each year using the R functions rbindlist from the data.table package to match each survey year on the corresponding variable columns. Only identical questions related to our research objectives were included in the combined data set.

All responses recorded as the option “not ascertained” were recoded as NA for all variables. All the variables were recoded by assigning binary values or group responses to facilitate the data analysis. The variables used as outcome variables were recoded to become binary variables: health information seeking using the internet (HIT1A), scheduling health care appointments using the internet (HIT3A), and communication with health care providers through email using the internet (HIT4A). Demographic variables were also recoded, including the respondent’s race (RACERPI2), Hispanic ethnicity (HISPANI_I), sex (SEX), region (REGION), marital status (R_MARITL), internet use (AWEBUSE) and the frequency of using the internet (AWEBOFNO and AWEBOFTP), email use (AWEBEML) and the frequency of using email (AWEBMTP), whether a respondent would go to a clinic or doctor’s office (AUSUALPL and APLKIND), and where a respondent goes to seek preventative care (AHCPLKND). Additionally, whether a respondent skipped medication doses to save money (ARX12_1), whether a respondent took less medication to save money (ARX12_2), if a respondent had delayed filling a prescription (ARX12_3), preferred low-cost medication (ARX12_4), reported buying prescription drugs from another country to save money (ARX12_5), used alternative therapies to save money (ARX12_6), affordability of prescribed medication (AHCAFYR1), affordability of mental health care or counseling (AHCAFYR2), affordability of dental care (AHCAFYR3), affordability of eyeglasses (AHCAFYR4), and worries about paying medical bills (AWORPAY).

All responses where the data were coded as “not ascertained” or “missing” were recoded as “NAs” for all of the variables and were dropped from the analysis. Whether or not a survey respondent had looked up health information using the internet in the last 12 months was recoded to a binary response of 1 to indicate if the respondent had looked up health information on the internet or 0 if they did not indicate looking up health information on the internet (all other responses). Similarly, all binary variables were recoded in the same manner. A survey respondent’s race was identified within the data by the RACERPI2 variable from the NHIS data. The variable was recoded to indicate if the survey respondent reported their race as being White, Black or African American, American Indian or Alaska Native, Asian, or multiple races. A survey respondent’s Hispanic ethnicity was recoded to indicate if the survey respondent reported being Hispanic, multiple Hispanic, Puerto Rican, Mexican, Mexican-American, Cuban or Cuban American, Dominican (Republic), Central or South American, other Latin American (type not specified), other Spanish, Hispanic or Latino or Spanish (nonspecific type), Hispanic or Latino or Spanish (type refused), or were not Hispanic (a response of 12, not Hispanic or Spanish origin). A survey respondent’s region was identified within the data using the variable REGION from the NHIS data. The variable was recoded to indicate if the survey respondent reported residing in the Northeastern, Midwestern, Southern, or Western United States. A survey respondent’s marital status was recoded to indicate if the survey respondent reported living with a spouse or partner or not living with a spouse or partner (all other responses). A survey respondent’s internet use was identified in the data using the AWEBUSE variable from the NHIS data. The variable was recoded to indicate if the survey respondent reported using the internet, or not. Additionally, a survey respondent’s internet use frequency was identified in the data using the AWEBOFNO and AWEBOFTP variables. The variables were recoded to indicate frequently using the internet (responses of once per day or more frequently) or not using the internet frequently (all other response combinations). The variable APLKIND was recoded to indicate if a respondent goes to a clinic or doctor’s office (a response of clinic or health center, or doctor’s office or health maintenance organization) or somewhere other than a clinic or doctor’s office (all other responses). These variables were combined to indicate if a respondent goes to a clinic or doctor’s office when sick, goes somewhere other than a clinic or a doctor’s office when sick, or does not indicate going anywhere when sick. Where a respondent goes to seek preventative care was identified using the variable AHCPLKND in the NHIS data. The variable was recoded to indicate if a respondent goes to a clinic or doctor’s office for preventative care (a response of clinic or health center, or doctor’s office or health maintenance organization) or does not go to a clinic or doctor’s office for preventative care (all other responses).

We used logit regression models to examine the relationship between our predictor variables and each of our 3 separate questions about the survey participants’ use of telehealth: looking up health information using the internet, scheduling an appointment with a health care provider using the internet, and communicating with a provider over email using the internet. In the field of machine learning and statistics, a wide range of computer models can be used for predicting clinical outcomes such as logit regression, decision trees, artificial neural networks, and Bayesian networks. We have selected Logit regression because it is a well-known statistical fitting model that is frequently used for modeling medical problems where it is needed to identify the relation between a binary response variable and a set of independent predictor variables [29]. The models were built using the svyglm function within the survey package in R version 4.2.1 (R Foundation for Statistical Computing) in R Studio 2023.03.0+386 (Posit PBC). The svyglm function includes the ability to account for a weighting variable in the data to facilitate population estimates. The stepAIC function within the MASS package was used to identify the best-fit model for this data.

## Results

### Overview

Young adults (18-35 years old) made up between 32.1% (n=10,140, unweighted) and 31.26% (n=6902, unweighted) of weighted survey respondents in 2012 and 2017, respectively, as shown in Table 1, while older adults (56-85 years old) accounted for 32.37% (n=12,662, unweighted) in 2012 and 35.63% (n=11,483, unweighted) in 2018. Women made up between 51.72% (n=13,867, unweighted) and 51.87% (n=19,252, unweighted) of the weighted survey respondents over the years.

**Table 1:** Demographic information broken down by year; given as count of response and percentage of weighted totals^a^.

Parameter			2012		2013		2014		2015	2016		2017		2018
**Age, n (%)**														
	Younger adult	10,140 (32.10)		10,183 (31.92)		10,431 (31.85)		9230 (31.73)		8662 (31.68)	6902 (31.26)		6177 (31.34)	
	Middle-aged	11,723 (35.53)		11,521 (35.31)		12,065 (34.64)		10,894 (34.23)		10,094 (33.60)	8176 (33.27)		7757 (33.02)	
	Older adult	12,662 (32.37)		12,853 (32.77)		14,201 (33.51)		13,548 (34.05)		14,272 (34.72)	11,664 (35.47)		11,483 (35.63)	
**Sex, n (%)**														
	Male	15,273 (48.13)		15,440 (48.16)		16,398 (48.20)		15,071 (48.20)		14,991 (48.23)	12,096 (48.24)		11,550 (48.28)	
	Female	19,252 (51.87)		19,117 (51.84)		20,299 (51.80)		18,601 (51.80)		18,037 (51.77)	14,646 (51.76)		13,867 (51.72)	
**Race, n (%)**														
	AIAN^b^	349 (0.82)		360 (0.83)		377 (0.81)		392 (0.95)		357 (1.01)	307 (1.20)		295 (1.13)	
	Asian	2183 (5.35)		2153 (5.59)		2129 (5.74)		1983 (5.94)		1670 (6.07)	1402 (6.37)		1350 (6.41)	
	Black or African American	5319 (11.91)		5361 (12.03)		5173 (12.26)		4673 (12.32)		3685 (12.31)	2980 (12.42)		2974 (12.38)	
	Multiple races	659 (1.67)		662 (1.57)		734 (1.61)		699 (1.74)		687 (1.93)	529 (1.99)		563 (2.32)	
	White	25,939 (80.25)		25,935 (79.98)		28,209 (79.57)		25,831 (79.05)		26,524 (78.68)	21,472 (78.93)		20,173 (77.75)	
**Region, n (%)**														
	Northeast	5774 (18.20)		5645 (17.52)		5919 (17.31)		5580 (17.45)		5590 (18.30)	4348 (18.31)		4143 (17.34)	
	Midwest	7193 (22.72)		7070 (22.68)		7809 (22.99)		7102 (22.42)		7345 (22.17)	6350 (21.81)		5949 (21.98)	
	South	12,536 (36.43)		12,813 (36.93)		12,896 (37.24)		11,646 (37.12)		11,487 (35.65)	9860 (36.21)		9312 (36.90)	
	West	9022 (22.65)		9029 (22.88)		10073 (22.46)		9344 (23.01)		8606 (23.88)	6184 (23.67)		6013 (23.78)	
Living with spouse or partner, n (%)			14,371 (48.15)		14,199 (48.27)		15,424 (48.08)		14,213 (48.11)	14,160 (48.07)		11,400 (48.66)		11,031 (48.89)
Unweighted sample size			34,525		34,557		36,697		33,672	33,028		26,742		25,417
Weighted sample size			234,920,670		237,394,354		239,688,457		242,500,657	245,142,225		246,657,271		249,455,533

^a^The table shows the unweighted sample size for each variable and the related percentage once the weights are applied to the data.

^b^AIAN: American Indian or Alaska Native.

The percentage of frequent internet users increased from 58.77% (n=18,016, unweighted) in 2012 to 73.15% (n=17,153, unweighted) in 2018. The percentage of email users increased from 64.46% (n=20,038, unweighted) in 2012 to 74.37% (n=17,635, unweighted) in 2018. The number of individuals who reported living with their spouse or partner ranged from 48.07% (n=14,160, unweighted) of weighted survey respondents in 2016 to 48.89% (n=11,031, unweighted) in 2018.

**Figure 1:**
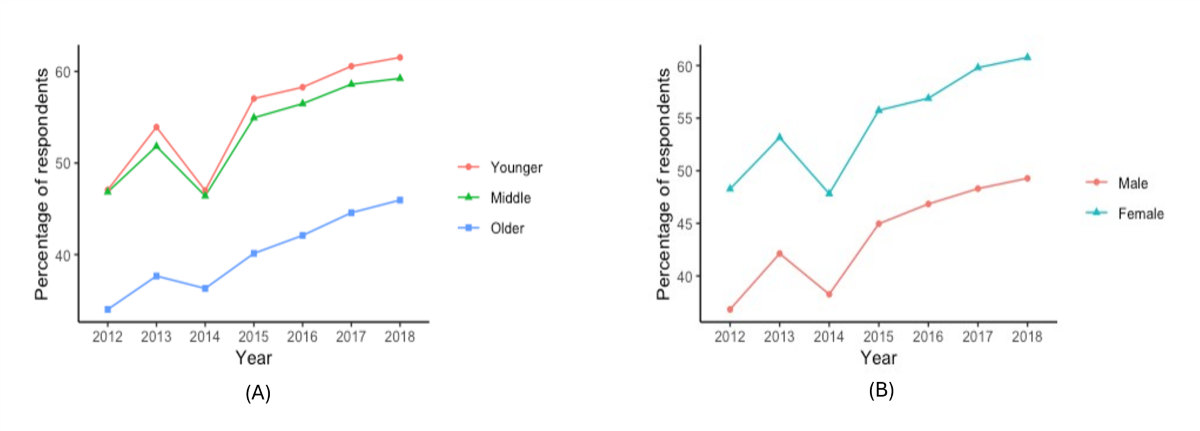
Percentage of respondents that looked up health information using the internet (A) for each age group and (B) by sex.

**Figure 2:**
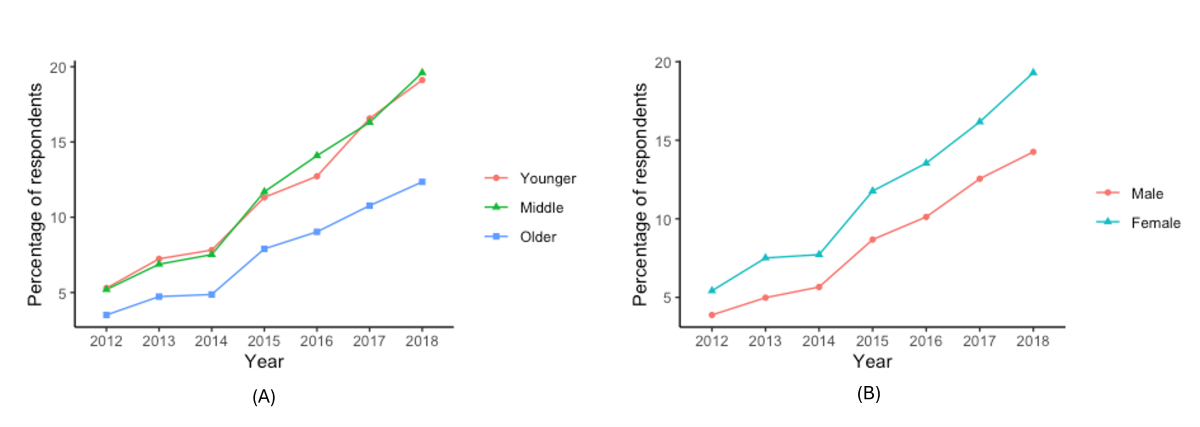
Percentage of respondents that scheduled an appointment using the internet (A) for each age group and (B) by sex.

**Figure 3:**
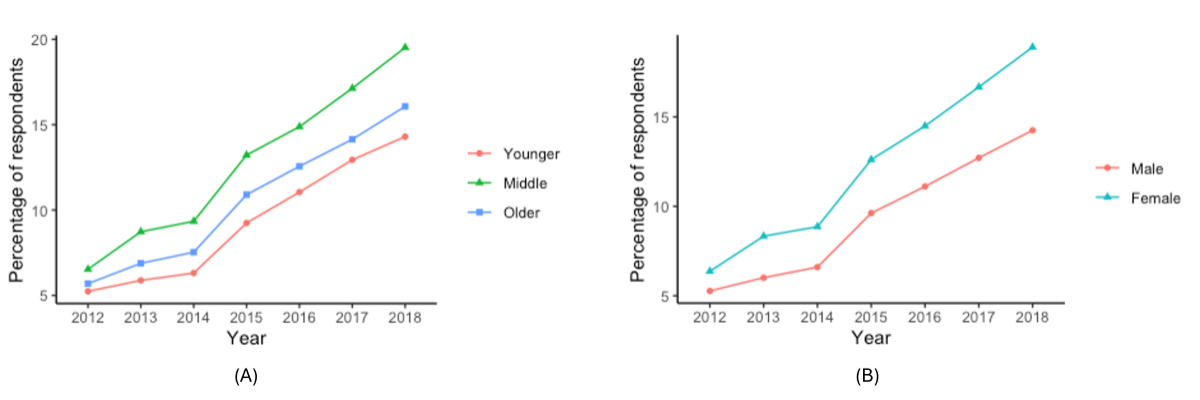
Percentage of respondents that emailed their care provider using the internet (A) for each age group and (B) by sex.

As shown in Figure 1A, older adult respondents tend to look up health information less than the middle-aged or younger adult groups over the years, while the percentage of the respondents looking up health information using the internet grew at similar rates for all groups. As shown in Figure 1B, men tend to look up health information using the internet less than women across all years of the study. Older adult respondents tended to report scheduling an appointment using less than younger adults or middle-aged respondents (Figure 2A). Across the years included in this study, the proportion of younger and middle-aged respondents tended to grow at similar rates. As shown in Figure 2B, men tended to report scheduling an appointment using the internet less often than women. As shown in Figure 3A, a higher percentage of middle-aged adults tended to report communicating with their care provider through email than older adults; a higher percentage of older adults tended to report it than younger adults. As shown in Figure 3B, men tended to report communicating with their care provider through email less often than women.

The number of respondents who accessed health information by using the internet increased from 42.76% (n=13,621, unweighted) in 2012 to 55.22% (n=13,677, unweighted) in 2018 (Table 2). In 2018, the percentage of respondents who scheduled an appointment using the internet rose from 4.68% (n=1463, unweighted) in 2012 to 16.86% (n=3962, unweighted). The number of respondents emailing their provider using the internet increased from 5.84% (n=1800, unweighted) in 2012 to 16.65% (n=4176, unweighted) in 2018. The number of respondents seeking medical care who visited a clinic or doctor’s office increased from 79.95% (n=27,085, unweighted) in 2012 to 82.03% (n=21,092, unweighted) in 2018. The number of respondents seeking preventive care who visited a clinic or doctor’s office increased from 35.96% (n=2706, unweighted) in 2012 to 45.16% (n=2133, unweighted) in 2018. The number of respondents concerned about paying medical bills decreased from 50.24% (n=17,267, unweighted) in 2012 to 43.54% (n=10,637, unweighted) in 2018. The use of alternative therapies to save money increased from 4.07% (n=1454, unweighted) in 2012 to 5.14% (n=1294, unweighted) in 2018.

**Table 2:** Response variables broken down by year; given as the count of responses and percentage of weighted totals^a^.

Parameter			2012		2013		2014		2015		2016		201		2018
Uses internet frequently (>daily), n (%)			18,016 (58.77)		19,699 (63.28)		21,463 (65.04)		19,380 (67.12)		21,013 (69.45)		17,596 (71.05)		17,153 (73.15)
Uses email, n (%)			20,038 (64.46)		21,255 (67.51)		22,697 (68.24)		20,008 (68.84)		21,873 (71.44)		18,198 (72.51)		17,635 (74.37)
Search health information using the internet, n (%)			13,621 (42.76)		15,241 (47.86)		14,783 (43.21)		15,917 (50.55)		16,543 (52.04)		14,097 (54.25)		13,677 (55.22)
Schedule appointment using the internet, n (%)			1463 (4.68)		1973 (6.29)		2099 (6.73)		2974 (10.28)		3542 (11.89)		3543 (14.42)		3962 (16.86)
Email care providers, n (%)			1800 (5.84)		2252 (7.21)		2550 (7.77)		3295 (11.17)		3994 (12.86)		3822 (14.76)		4176 (16.65)
Skip medication to save money, n (%)			2293 (6.31)		1745 (7.72)		1654 (6.93)		1400 (6.07)		1340 (5.78)		1071 (5.96)		1003 (5.71)
Take less medication to save money, n (%)			2418 (6.67)		1875 (8.21)		1733 (7.21)		1519 (6.46)		1390 (6.01)		1118 (6.08)		1074 (5.95)
Request lower-cost medication, n (%)			6320 (18.72)		5186 (24.39)		4918 (21.35)		4259 (19.45)		4460 (19.65)		3456 (18.74)		3436 (19.20)
Uses international medications to save money, n (%)			663 (1.90)		588 (1.57)		554 (1.45)		483 (1.31)		477 (1.62)		397 (1.51)		388 (1.58)
Alternate therapies to save money, n (%)			1454 (4.07)		1559 (4.20)		1587 (4.04)		1358 (3.78)		1411 (4.26)		1161 (4.20)		1294 (5.14)
Cannot afford medications, n (%)			3040 (8.29)		2838 (7.81)		2623 (6.89)		2243 (6.35)		2151 (6.19)		1661 (6.07)		1689 (6.30)
Cannot afford mental care, n (%)			944 (2.51)		798 (2.10)		766 (1.92)		682 (1.87)		686 (1.95)		582 (2.11)		665 (2.65)
Cannot afford dental care, n (%)			4776 (13.23)		4748 (12.85)		4490 (11.60)		3913 (10.90)		3498 (10.24)		2891 (10.77)		2922 (11.26)
Cannot afford vision care, n (%)			2860 (7.80)		2734 (7.33)		2574 (6.47)		2398 (6.40)		1971 (5.64)		1607 (5.81)		1676 (6.33)
Worried about paying medical bills, n (%)			17,237 (50.24)		17,106 (49.76)		16,912 (46.87)		15,136 (45.55)		13,816 (43.72)		11,296 (44.44)		10,637 (43.54)
**Goes to a clinic when sick, n (%)**															
	No where	5660 (16.17)		5325 (15.27)		5033 (13.69)		4518 (13.80)		3941 (13.07)		3167 (12.98)		3137 (13.69)	
	Somewhere else	1442 (3.88)		1551 (3.90)		1543 (3.97)		1304 (3.59)		1108 (3.27)		947 (3.48)		1047 (4.29)	
	Goes to clinic	27,085 (79.95)		27,486 (80.83)		29,841 (82.34)		27,587 (82.60)		27,757 (83.66)		22,452 (83.54)		21,092 (82.03)	
Visits clinic for preventive care			2706 (35.96)		2478 (33.78)		2607 (35.74)		2495 (39.04)		2640 (44.76)		2191 (46.10)		2133 (45.16)
Unweighted sample size			34,525		34,557		36,697		33,672		33,028		26,742		25,417
Weighted sample size			234,920,670		237,394,354		239,688,457		242,500,657		245,142,225		246,657,271		249,455,533

^a^The table shows the unweighted sample size for each variable and the related percentage once the weights are applied to the data.

### Looking Up Health Information Using the Internet

We constructed a model to examine the relationship between the predictor variables and whether or not the survey respondent accessed health information using the internet (Table 3). With each successive year, respondents were more likely to look up health information using the internet (odds ratio [OR] 1.12, 95% CI 1.10-1.15). Middle-aged adults (OR 0.83, 95% CI 0.75-0.93) and older adults (OR 0.65, 95% CI 0.57-0.73) were less likely in comparison to younger adults to look up health information using the internet. Women were more likely to look up health information using the internet in comparison to men (OR 1.69, 95% CI 1.54-1.85). Black or African American respondents were less likely to look up health information in comparison to White respondents (OR 0.77, 95% CI 0.67-0.89). The survey respondents in the South region were less likely to look up health information using the internet compared to those in the North region (OR 0.73, 95% CI 0.63-0.85). A frequent internet user was more likely to look up health information (OR 3.40, 95% CI 3.00-3.85). Similarly, respondents who used email regularly were more likely to look up health information using the internet than those who reported not using email frequently (OR 3.88, 95% CI 3.41-4.42). Respondents who reported asking their clinicians for lower-cost medications in order to save money were more likely across all years to look up health information (OR 1.55, 95% CI 1.38-1.75). Respondents using alternative therapies in order to save money were almost twice as likely to look up health information using the internet (OR 1.92, 95% CI 1.57-2.36). Respondents who reported going to a clinic or doctor’s office when they were sick were more likely to look up health information using the internet than those who did not report going there (OR 1.21, 95% CI 1.08-1.36). Respondents who reported going to a clinic or doctor’s office as part of preventative care were also more likely to look up health information using the internet than those who did not seek out preventative care at a clinic or doctor’s office (OR 1.18, 95% CI 1.07-1.31).

**Table 3:** The best-fit model predicting the likelihood that an individual look up health information using the internet.

Parameter	Parameter estimate (SE)	*t* test	*P* value	OR^a^ (95% CI)
Intercept	–238.10 (21.92)	–10.86	<.001	N/A^b^
Year	0.12 (0.01)	10.77	<.001	1.12 (1.10-1.15)
Middle-aged	–0.18 (0.05)	–3.35	<.001	0.83 (0.75-0.93)
Older adult	–0.44 (0.06)	–6.81	<.001	0.65 (0.57-0.73)
Female	0.52 (0.05)	11.25	<.001	1.69 (1.54-1.85)
Black or African American	–0.26 (0.07)	–3.62	<.001	0.77 (0.67-0.89)
AIAN^c^	–0.32 (0.24)	–1.33	.18	NS^d^
Asian	–0.28 (0.11)	–2.64	.008	NS
Multiple race	0.00 (0.14)	0.03	.98	NS
Midwest	–0.14 (0.08)	–1.65	.10	NS
South	–0.31 (0.076)	–4.16	<.001	0.73 (0.63-0.85)
West	–0.11 (0.08)	–1.31	.19	NS
Not living with spouse	–0.15 (0.049)	–3.10	.002	NS
Uses internet frequently	1.23 (0.06)	19.29	<.001	3.40 (3.00-3.85)
Uses email	1.36 (0.07)	20.60	<.001	3.88 (3.41-4.42)
Used lower-cost medication to save money	0.44 (0.06)	7.19	<.001	1.55 (1.38-1.75)
Used drugs from other countries to save money	–0.30 (0.15)	–1.95	.05	NS
Used alternate therapies to save money	0.65 (0.10)	6.28	<.001	1.92 (1.57-2.36)
Cannot afford mental care	0.26 (0.11)	2.46	.01	NS
Cannot afford dental care	0.20 (0.07)	2.88	.004	NS
Cannot afford eye care	0.12 (0.08)	1.44	.15	NS
Worried about paying for medical bills	0.08 (0.05)	1.70	.09	NS
Goes somewhere other than a clinic or doctor’s office when sick	0.13 (0.07)	1.82	.07	NS
Goes to a clinic or doctor’s office when sick	0.19 (0.05)	3.36	<.001	1.21 (1.08-1.36)
Goes to a clinic or doctor’s office for preventative care	0.17 (0.05)	3.39	<.001	1.18 (1.07-1.31)

^a^OR: odds ratio.

^b^N/A: not applicable.

^c^AIAN: American Indian or Alaska Native.

^d^NS: parameter was not significant at α=.001.

### Scheduling an Appointment Using the Internet

We constructed a model to examine the relationship between the predictor variables and whether or not the survey respondent scheduled an appointment using the internet (Table 4). With each successive year, respondents were more likely to schedule an appointment using the internet (OR 1.30, 95% CI 1.26-1.34). Older adults were less likely in comparison to younger adults to schedule an appointment using the internet (OR 0.63, 95% CI 0.51-0.78). Women were more likely than men to schedule an appointment using the internet (OR 1.61, 95% CI 1.40-1.85). Respondents who use the internet frequently were more likely to schedule an appointment than those not using the internet frequently (OR 2.75, 95% CI 2.02-3.74). Survey respondents using email were more likely than respondents not using email to schedule an appointment using the internet (OR 3.92, 95% CI 2.78-5.53). Respondents who used alternative therapies in order to save money were more likely to schedule an appointment using the internet than those who did not report using alternative therapies to save money (OR 1.70, 95% CI 1.34-2.16). Respondents who were worried about paying medical bills in the last 12 months were less likely than those who did not report being worried about paying medical bills to schedule an appointment using the internet (OR 0.77, 95% CI 0.66-0.89). Respondents going to a clinic or doctor’s office for preventative care were more likely to schedule an appointment using the internet than those who did not report going to a clinic or doctor’s office for preventative care (OR 1.55, 95% CI 1.33-1.82).

**Table 4:** The best-fit model predicting the likelihood that an individual schedules a health care appointment using the internet.

Parameter	Parameter estimate (SE)	*t* test	*P* value	OR^a^ (95% CI)
Intercept	–537.38 (32.31)	–16.63	<.001	N/A^b^
Year	0.26 (0.02)	16.49	<.001	1.30 (1.26-1.34)
Middle-aged	–0.19 (0.08)	–2.37	.02	NS^c^
Older adult	–0.46 (0.11)	–4.22	<.001	0.63 (0.51-0.78)
Female	0.48 (0.07)	6.63	<.001	1.61 (1.40-1.85)
Black or African American	–0.08 (0.11)	–0.66	.51	NS
AIAN^d^	–0.71 (0.53)	–1.34	.18	NS
Asian	0.40 (0.14)	2.88	.004	NS
Multiple race	0.20 (0.23)	–0.89	.37	NS
Midwest	–0.37(0.12)	–3.01	.003	NS
South	–0.10 (0.11)	–0.92	.36	NS
West	0.03 (0.11)	0.26	.79	NS
Not living with spouse or partner	–0.08 (0.07)	–1.10	.28	NS
Uses internet frequently (>1 per day)	1.01 (0.16)	6.46	<.001	2.75 (2.02-3.74)
Uses email	1.37 (0.18)	7.80	<.001	3.92 (2.78-5.53)
Used lower-cost medication to save money	0.26 (0.09)	2.96	.003	NS
Used alternate therapies to save money	0.53 (0.12)	4.39	<.001	1.70 (1.34-2.16)
Cannot afford prescription medicine	–0.23 (0.14)	–1.67	.09	NS
Cannot afford mental care	0.37 (0.17)	2.21	.03	NS
Cannot afford dental care	–0.28 (0.11)	–2.40	.02	NS
Worried about paying for medical bills	–0.26 (0.08)	–3.46	<.001	0.77 (0.66-0.89)
Goes somewhere other than a clinic or doctor’s office when sick	–0.07 (0.12)	–0.55	.58	NS
Goes to a clinic or doctor’s office when sick	0.18 (0.08)	2.16	.03	NS
Goes to a clinic or doctor’s office for preventative care	0.44 (0.08)	5.50	<.001	1.55 (1.33-1.82)

^a^OR: odds ratio.

^b^N/A: not applicable.

^c^NS: parameter was not significant at α=.001.

^d^AIAN: American Indian or Alaska Native.

### Communicating With a Care Provider Through Email Using the Internet

We constructed a model to examine the relationship between the predictor variables and whether or not the survey respondent communicated with a care provider through email using the internet (Table 5). With each successive year, respondents were more likely to communicate with a care provider through email using the internet (OR 1.24, 95% CI 1.20-1.28). Women were more likely than men to communicate with a care provider through email using the internet (OR 1.48, 95% CI 1.28-1.71). Respondents who use the internet frequently were more likely to communicate with a care provider through email than those not using the internet frequently (OR 2.08, 95% CI 1.56-2.76). Survey respondents using email were more likely than respondents not using email to communicate with a care provider through email (OR 5.56, 95% CI 3.84-8.05). Respondents who could not afford mental care in the last 12 months were more likely to communicate with a care provider through email using the internet than those who could afford mental care in the last 12 months (OR 1.92, 95% CI 1.39-2.66). Respondents who reported going to a clinic or doctor’s office when they were sick were more likely to communicate with a care provider through email using the internet than those who did not report going there when sick (OR 1.32, 95% CI 1.12-1.55). Respondents who reported going to a clinic or doctor’s office as part of preventative care were also more likely to communicate with a care provider through email using the internet than those who did not seek out preventative care at a clinic or doctor’s office (OR 1.57, 95% CI 1.34-1.85).

**Table 5:** The best-fit model predicting the likelihood that an individual communicated with a care provider through email using the internet.

Parameter	Parameter estimate (SE)	*t* test	*P* value	OR^a^ (95% CI)
Intercept	–442.69 (32.92)	–13.45	<.001	N/A^b^
Year	0.22 (0.02)	13.29	<.001	1.24 (1.20-1.28)
Middle-aged	0.16 (0.08)	1.88	.06	NS^c^
Older adult	0.18 (0.11)	1.71	.09	NS
Female	0.39 (0.07)	5.34	<.001	1.48 (1.28-1.71)
Black or African American	0.02 (0.13)	0.14	.89	NS
AIAN^d^	–0.37 (0.42)	–0.90	.37	NS
Asian	0.37 (0.15)	2.50	.01	NS
Multiple race	–0.44 (0.24)	–1.80	.07	NS
Midwest	–0.09 (0.13)	–0.72	.47	NS
South	–0.09 (0.12)	–0.79	.43	NS
West	0.32 (0.12)	2.71	.007	NS
Not living with spouse or partner	–0.19 (0.07)	–2.55	.01	NS
Uses internet frequently (>1 per day)	0.73 (0.14)	5.04	<.001	2.08 (1.56-2.76)
Uses email	1.72 (0.19)	9.10	<.001	5.56 (3.84-8.05)
Skipped medication to save money	0.31 (0.20)	1.55	.12	NS
Took less medication to save money	–0.30 (0.21)	–1.43	.15	NS
Used lower-cost medication	0.30 (0.09)	3.18	.002	NS
Used alternate therapies	0.41 (0.13)	3.11	.002	NS
Cannot afford medication	–0.24 (0.16)	–1.50	.13	NS
Cannot afford mental care	0.65 (0.16)	3.98	<.001	1.92 (1.39-2.66)
Cannot afford dental care	–0.30 (0.13)	–2.26	.02	0.74 (0.57-0.96)
Cannot afford eye care	–0.19 (0.17)	–1.14	.26	NS
Worried about a medical bill	–0.18 (0.08)	–2.35	.02	NS
Goes somewhere other than a clinic or doctor’s office when sick	0.10 (0.12)	0.85	.39	NS
Goes to a clinic or doctor’s office when sick	0.28 (0.08)	3.32	<.001	1.32 (1.12-1.55)
Goes to a clinic or doctor’s office for preventative care	0.45 (0.08)	5.50	<.001	1.57 (1.34-1.85)

^a^OR: odds ratio.

^b^N/A: not applicable.

^c^NS: parameter was not significant at α=.001.

^d^AIAN: American Indian or Alaska Native.

## Discussion

### Principal Findings

This study used NHIS data, which is weighted to represent national characteristics, to examine the use of technology for performing some health care–related tasks over time: looking up health information using the internet, scheduling an appointment using the internet, and communicating with a care provider over email using the internet. Across all our models, we found that middle-aged and older adult respondents were significantly less likely to use technology to look up health information using the internet or schedule an appointment using the internet versus younger individuals. It has been shown that some older adults may need additional special usability requirements due to their inexperience in the use of technology [21], yet this population is a growing user of technology [30]. This also reaffirmed the results found in the existing literature [26]. Across all of the variables we investigated, we found that the rates of looking up health information using the internet, scheduling an appointment using the internet, and communicating with a care provider over email using the internet increased substantially across the study period. This demonstrates that there is an increasing use and need for these services to support larger populations of users including devices and abilities to engage with technology.

Specifically, our analysis found that middle-aged adults were found to have a higher OR than older adults (0.83 vs 0.65) for looking up health information using the internet. We also found that there were differences in age groups for using technology to perform health care–related tasks. In terms of searching health information and scheduling appointments using the internet, we found differences between men and women, with women being significantly more likely than men to look up health information using the internet, schedule an appointment using the internet, and communicate with a care provider through email using the internet. There are conflicting results in the literature related to sex differences in the use of the internet and technology associated with health-related tasks. Newhouse et al [31] found that men were more likely to use email communication for health care than women; however, Baumann et al [32] found that women were more likely to use the internet and technology for health-related tasks such as scientific literature review, communicating with their physician or medical team, and interpreting the diagnostic test results and medications used in treatment. These sex differences may be related to the social construction and perceptions of technology [33]. Future research needs to explore why these trends are occurring and what factors are associated with the differences between men and women especially since there are conflicting results in the literature about use but also about the factors associated with the differences in use.

The impact of costs was inconsistent across the different models in our analysis. With respect to looking up health information using the internet, there was a significant association with using lower-cost medications and alternative therapies to save money. For individuals scheduling a health care appointment using the internet, respondents who indicated using alternative therapies to save money were more likely to schedule appointments using the internet, and surprisingly those who indicated being worried about paying for medical bills were less likely to schedule appointments using the internet. Individuals using email to communicate with a care provider had two different cost-related variables that were significant. Respondents who reported not being able to afford mental care were more likely to email, while those who indicated not being able to afford dental care were less likely to email. In some ways, the included cost-related variables did not seem to indicate a consistent response around cost. This reinforces the notion that determining the extent to which the cost of care is a barrier is challenging and unclear [34]. By examining the results across several cost-of-care–related variables and different elements of health care, it appears that there may be other barriers whose interaction with cost impacts the effect of cost as a barrier. As suggested by Clarke et al [34], and supported by our results, telehealth research should continue to investigate the ways in which different types of costs are intertwined with the use of telehealth; especially as it relates to different avenues of care.

Going to a clinic or doctor’s office for preventative care was associated with a greater likelihood of looking up health information using the internet, schedule a health care appointment using the internet, and communicate with a care provider through email using the internet. Going to a clinic or doctor’s office when sick was associated with using technology to look up health information and communicating with a care provider through email. This suggests that there is a strong correlation between respondents’ collaboration in their personal health and the likelihood that they would use telehealth services to meet these needs. In fact, Sawesi et al [35] found that information technology platforms can enhance patient engagement and improve health outcomes. It is crucial that telehealth research investigate ways in which telehealth can be used to either support individuals in developing an engagement in their own health or identify ways to encourage users to develop an engagement in their own health.

### Limitations and Future Work

There are several factors that limit the generalizability of our analyses. One notable limitation of this research is that it relies on self-reported survey data and may not fully capture specific perspectives and opinions about the “why” for some of the activities reported in the NHIS. Yet, the NHIS data collected and maintained by the US CDC is widely used [36-38] and the data, the data design, and the imputation for national representation are widely documented [39,40]. Future research should expand on these results to try to identify if these trends continue or what factors may be driving the differences identified over time.

This research uses responses from specific questions from 2012 to 2018, as those were the only consecutive years where these questions were consistent. As a result, being able to project current and future use of technology for telehealth is limited. Additionally, the COVID-19 pandemic had a major impact on health care as it presented a unique situation that resulted in overcoming many of the traditional barriers to telehealth adoption [41]. COVID-19 also emphasized the use cases of a variety of different technological tools that can be used in telehealth such as chatbots [42]. As our data was limited to data collected before the COVID-19 pandemic, it is important that future work examine the impact that the pandemic has had on the use of telehealth services and if these effects have been sustained in the following years. Future research should also investigate the ways in which the pandemic might have impacted or altered the trends and barriers examined in this research. For example, it may be interesting to evaluate if the exposure and use of these tools during the pandemic have had lasting impacts on the use of the internet tools. To do this, we need similar data from 2020-2023 or later from the NHIS on the use of telehealth services to be able to apply time series modeling techniques to evaluate specific trends in how using telehealth services have changed over time. Interestingly, some initial work has suggested that there is a decline in the use of telemedicine between 2021 and 2022 in the United States, [43] and future work should evaluate if that trend continues or if the trend prior to the COVID-19 pandemic continues.

In order to develop a fundamental understanding of the important barriers, this research did not examine the complex relationships between demographic and response variables. Future research should focus on understanding how these interactions (eg, older women vs middle-aged women vs younger women) might be correlated with the respondents’ use of telehealth. Our models also did not investigate telehealth use for specific socioeconomic levels. Instead, it includes questions regarding a respondent’s ability to afford different health care services, which is different from financial security or socioeconomic conditions [44].

### Conclusions

As telehealth is increasingly becoming an important component of health care services, it is important to focus on different aspects of telehealth to determine the demographic characteristics, behaviors, or opinions that may predict or influence groups that are likely to face a barrier to using telehealth services. This study used NHIS data to examine the use of technology for performing some health care–related tasks over time: looking up health information using the internet, scheduling an appointment using the internet, and communicating with a care provider over email using the internet. From this analysis, we have found some potential barriers that may impact a user’s ability to access telehealth services, as well as differences in the use of these tools for different groups. Understanding those who are using the internet for health care–related activities and the barriers that they may face is important for the design and implementation of these systems to be as effective as possible. Systems and technology designers, as well as health care providers, should be aware of these differences and the impacts they may have on engaging individuals in their health care.

## Abbreviations

CDC: Centers for Disease Control and Prevention
NHIS: National Health Interview Survey
OR: odds ratio
